# Weak Food

**Published:** 1875-11

**Authors:** 


### WEAK FOOD.

We have all been badly educated in regard to the appearance which certain foods should present to the sense of sight. We have been taught to believe that white bread, for example, is better than dark. This is a grievous error. The white part of wheat is its weakest part. It is nearly all starch, and starch, when assimilated by digestion, is only sugar—a warming food,—but not a giver of strength, a repairer of waste places. Starch, whether found in the potato or in wheat or corn, is probably our most useless food; yet thousands and millions depend upon it chiefly for support.

Let us not be misunderstood. Starch is all well enough when adequately mixed with more nutritious food. It is not to be despised or avoided when employed in proper proportions and in a proper way. We would not have it taken out of our breadstuff, any more than we would have our bread wholly made from it, as most people do. We prefer to have it commingled with other and stronger elements, in the proportion fixed upon by nature and secured by cultivation. We do not consider it profitable to eat bread from which nearly all the real building-up particles have been carefully withdrawn merely to please somebody's vitiated eyesight. We want our bread to be genuine FOOD, and that is clearly outside the province of most bread now eaten in America.

The time was when bread was the "staff of life." It should be the strong support of humanity now. It is vastly more nutritious than meat, pound for pound, if rightly made, and is entirely adequate to support life, unaided by other foods. Good bread in abundance, eaten with fruit, affords all the strength of body and brain needed by mortal man in any climate. Only in the frigid zone is aught else demanded; and there the fats and starches find their appropriate place as heat-givers.

We sat down to a well spread dinner-table the other day, and at its close we wondered how sensible people could seriously make up their minds that such food as was there served was sufficient to sustain active and vigorous life. There was a thin soup—pleasant enough, but containing the scantiest amount of nourishment. Then followed roast beef and mutton pot-pie. The beef was good of its kind; but it would take a very large quantity of beef to repair the waste of a long day's labor, if indeed any amount of beef *alone* could do it. As for the mutton pot-pie, its leading feature was the great dumplings, or lumps of dough made of refined flour. Then followed pie and corn-starch pudding. The bread was nice, white, spongy, worthless baker's bread. Then there were sweet potatoes and Irish potatoes, and butter. The dinner was chiefly heat-producing, and contained only a trace of muscle-sustaining nutriment. It was a meal well calculated to induce dyspepsia, for a number of reasons. The flour-balls could not be readily digested, and the starch of these, as well as of the white flour bread, the potatoes, the pie, and the corn-starch, would tax the gastric juice of the most powerful. Yet it was "a good dinner" to the average man, and doubtless satisfactory to nearly all who partook of it.

Food like this, if taken daily, must weaken and destroy both body and brain. We could not live on it and be well, or do well. We should retire at night exhausted, and wake up languid.

How could we improve upon that meal? In this way. Wherever white flour entered into it, we would have substituted flour made from the best grain, without bolting or sifting. This would have given us gluten for the muscles, phosphorus for the brain, and lime for the bones. Instead of the pie and corn-starch at the end, we would have had fruit; or, perhaps, better still, nothing. This would have given us a good meal to work on, to digest, and to sleep soundly and sweetly upon.

By and by the world will learn that in bolting its cereals, in sifting out all that is not white, it robs itself of its real food. It is hard to understand how this ruinous practice has been brought about; but we apprehend that to the bakers we owe the practical destruction of our most valuable food. They have educated the people up to the idea that bread must be white to be good, when the truth is that the whiter the bread the more worthless it is. There is more solid nourishment in four good loaves of sweet unbolted wheat bread, made from plump grain, full of gluten, than in a cartload of bakers' snow-white trash, which has been bleached and doctored with ammonia and alum, to please disordered eyes and abnormal tastes.

---

Do away with female modesty, delicacy, and refinement of manners, and what remains lovely or prepossessing?

